# Considerations for Program Managers to Improve Sexual and Reproductive Health Services for Displaced Populations

**DOI:** 10.9745/GHSP-D-23-00036

**Published:** 2023-08-28

**Authors:** Patricia Doherty, Erin Wheeler, Vernon Mochache, Taban John Mark, Geoffrey Luttah, Berhanu Bero, Ellen Clancy, Melissa Cockroft, Ahmed Omar, Ginny Robins, Karine Penrose-Theis, Alexandra Todd

**Affiliations:** a Options Consultancy Services, London, United Kingdom.; b International Rescue Committee, New York, NY, USA.; c International Planned Parenthood Federation, Nairobi, Kenya.; d International Planned Parenthood Federation, Juba, South Sudan.; e International Rescue Committee, Nairobi, Kenya.; f MSI Reproductive Choices, Addis Ababa, Ethiopia.; g MSI Reproductive Choices, London, United Kingdom.; h Options Consultancy Services, Nairobi, Kenya.; i Options Consultancy Services, Washington, DC, USA.

## Abstract

Delivering sexual and reproductive health services to migrating and displaced populations is complex. The authors provide 4 recommendations drawing on their experience across Africa and Asia.

## Key Messages

- Displaced populations face profound challenges in accessing high-quality sexual and reproductive health and family planning services.
- As the drivers of displacement increase, driven by conflict, climate change, and humanitarian crises, the need for effective understanding and implementation of sexual and reproductive health and rights is ever more acute.
- We summarize key program insights and lessons focused on the need to strengthen country ownership, ensure a coordinated and adaptive approach, prioritize rights, and expand self-care to reach the most marginalized groups.

## BACKGROUND

Across the world, individuals make choices every day to further their opportunities and/or protect themselves and their families. This has always included migration, both across borders and within countries. The 2022 World Migration Report estimated that there were approximately 281 million international migrants in 2020.^1^ However, the majority of people migrate within countries. In 2013, the number of internal migrants totaled 763 million,^2^ and in 2020 alone, it was estimated that 55 million people were forcibly displaced within borders, with 87% of these displaced by conflict and the remaining 13% due to natural disasters.^1^ The number of people migrating is expected to increase, with conflicts, poor economic opportunities, and climate change motivating movement or hastening displacement.^3^ The 2022 Groundswell report estimated that climate change alone could force 216 million people across 6 world regions to move within their countries by 2050.^4^

Migration can lead to reduced access to health and social services, worse economic and social outcomes (including discrimination and social stigma), lack of legal entitlements, and administrative barriers. These challenges, coupled with cultural and linguistic barriers that migrants may face, can contribute to health inequities, increased health risks, and negative health outcomes.^5^ These effects are particularly acute among populations that have been forcibly displaced, with the majority hosted in low- and middle-income countries that may be facing their own humanitarian and global health challenges, including food crises, climate crises, extreme poverty, and disease outbreaks.^6^ In such situations, mechanisms for service delivery in areas such as health and education can be disrupted, and community and protection mechanisms can break down.

Displaced women and girls are particularly vulnerable to sexual violence, exploitation, and gender-based violence (GBV) during their journeys and in host communities.^7^ However, the provision of high-quality sexual and reproductive health (SRH) services to these populations can be limited both within stable host countries^7^ and in displaced settings. In addition, the challenges of conducting research among these populations have led to limited evidence on the impact of different interventions.

There is a continued need to strengthen knowledge and capacity around standards for global SRHR and work across the humanitarian/development nexus to ensure no one is left behind.

The *Inter-Agency Field Manual on Reproductive Health in Humanitarian Settings*^8^ and the *Minimum Initial Service Package (MISP) for SRH*, which is integrated into the *Sphere Handbook*,^9^ provide standards and recommendations for the application and adaptation of global sexual and reproductive health and rights (SRHR) in humanitarian settings. The MISP prioritizes lifesaving activities to be implemented at the onset of a crisis and aims to prevent mortality, morbidity, and disability in crises-affected populations by (1) ensuring there is a lead organization for implementing the MISP, (2) preventing sexual violence and responding to the needs of survivors, (3) preventing the transmission of and reducing morbidity and mortality due to HIV and other sexually transmitted infections (STIs), (4) preventing excess maternal and newborn morbidity and mortality, (5) preventing unintended pregnancy, and (6) planning for comprehensive SRH services integrated into primary health care as soon as possible.^10^

There is a continued need to strengthen knowledge and capacity around these standards and work across the humanitarian/development nexus to ensure no one is left behind. This commentary is a direct response to this call to action, recognizing and aiming to act immediately on the need to share practices and lessons. We draw on examples from the Women’s Integrated Sexual Health (WISH) program, which aims to improve the delivery of integrated SRH and family planning (FP) services (including STIs, sexual violence and GBV counseling, antenatal care, and postabortion care in line with local legal frameworks), supports individual knowledge and choice, and strengthens national stewardship to create an enabling and sustainable environment for advancing SRHR and FP. The program focuses on the most vulnerable and underserved populations, including people who have disabilities, have been displaced or affected by crisis, or are living in hard-to-reach areas.

Funded by UK aid, the program is implemented under 2 arms led by the International Planned Parenthood Federation and MSI Reproductive Choices (MSI). Implementing partners include Options Consultancy Services, which leads the national stewardship and enabling environment component; Development Media International, which leads demand creation; the International Rescue Committee, which primarily focuses on ensuring access to high-quality SRH services in humanitarian and fragile settings; and Humanity Inclusion, Leonard Cheshire, ThinkPlace, and Sightsavers, which ensure nondiscrimination and disability inclusion are mainstreamed across the program. The program prioritizes operational research, led and commissioned by the implementing partners. In addition, UK aid, in partnership with a third-party monitor, Itad, and Oxford Policy Management, regularly monitors and evaluates the implementation progress and results of the program, ensuring insights are given to implementing partners and UK aid to maximize performance and inform ongoing program learning and dissemination. WISH started in 2018 and has been implemented across 26 countries in sub-Saharan Africa and Asia. The current program is due to end in Spring 2024.

## RECOMMENDATIONS TO SUPPORT DELIVERY OF HIGH-QUALITY SERVICES IN CRISES SITUATIONS

We share 4 recommendations gleaned from operational program experience over the past 5 years on how to support the delivery of high-quality and accessible SRH and FP services in situations of crisis and/or for migrant populations. In this commentary, we refer to refugee populations and internally displaced groups who have been forcibly displaced either by conflict or natural disaster.

### 1. Strengthening Country Ownership for High-Quality, Well-Financed, Respectful, and Accessible Services Is Essential

Health systems and plans must be responsive to the needs of the populations they serve, including refugee populations and those who have been displaced. However, this is often not the case. The United Nations High Commission for Refugees Inclusion of Refugees into National Health System Survey found that of the 47 refugee hosting countries with national health plans or policies, only 29 included refugees within these plans or policies.^11^ As a result, these populations are not prioritized, and there can be confusion around who is accountable for delivering health services to them, with gaps and lack of coordination common, particularly around SRH and FP. This is true even in high-income stable contexts, as discussed in Hadgkiss and Renzaho’s systematic review, which looked at health access and barriers to care among those seeking asylum while living in host communities.^12^ The review found consistently lower preventive health service use among those seeking asylum compared to the host population.^12^

Across sub-Saharan Africa and Asia, countries are devolving and decentralizing. Within these settings, local governments vary in terms of how they consider and provide services to internally displaced populations. For example, in some parts of Ethiopia, cultural and ethnic differences can prevent displaced communities from accessing and being provided with high-quality SRH/FP public services. As a result, United Nations agencies and humanitarian organizations continue to play a significant role in meeting the SRH and FP needs of these groups.

Focusing on strengthening resilient national and subnational health systems is critical to responding to the evolving needs of displaced populations and preventing overreliance on humanitarian partners and donor-funded initiatives. The *Family Planning in Humanitarian Settings: A Strategy Guide*^13^ and the *FP 2030 Ready to Save Lives Preparedness Toolkit*^14^ provide national and subnational decision-makers with key actions that can be taken in the preparedness, response, and transition phases of crises. These actions have a core focus on supporting government and locally owned structures, including civil society. Key recommendations include ensuring appropriate policies are designed to minimize disruption to FP during a crisis, positioning the current workforce to facilitate access, leveraging routine systems for the efficient use of resources, and supporting the government to reestablish routine service delivery in affected areas.^14^

Focusing on strengthening resilient national and subnational health systems is critical to responding to the evolving needs of displaced populations and preventing overreliance on humanitarian partners and donor-funded initiatives.

Similarly, the *Inter-Agency Field Manual*^8^ emphasizes the importance of involving the community in the provision of services and working in respectful partnership with local actors. At the outset of a crisis, the health sector must identify a lead SRH organization that has the capacity to coordinate technical and operational support, facilitate coordinated action, identify implementation issues, work across clusters to roll out appropriate responses, share information, and ensure the community is aware of the availability and location of SRH services. This lead organization could be a ministry of health, United Nations agency, or nongovernmental organization and must allow for respectful and meaningful engagement with a diversity of perspectives, promote coordination, and use culturally sensitive approaches.

The program has worked in line with these critical principles, supporting government, civil society, and health providers to create a stronger enabling environment for SRHR through the implementation of evidence-based and well-financed policies that address the needs of women and girls and the creation of accountability systems and participatory decision-making spaces. For example, in South Sudan, the program supported the Directorate of Reproductive Health at the Ministry of Health in meetings with partners to discuss SRHR activities and progress across the country (including services for displaced populations) and in the coordination of the support needed for the delivery of services from relevant ministries and implementing partners. Although this effort may seem simple, ensuring that the government rather than an implementing partner owns and drives the coordination process is a critical step to ensuring stewardship of SRH/FP services is led and sustained by country actors rather than external and donor-funded initiatives. This level of country-led cross-sectoral collaboration has also been critical to mitigating threats that SRH/FP providers face from communities in South Sudan, which has constituted a significant challenge and risk.^15^ To date, this approach has helped contribute to 95,244 couple-years of protection^16^ and 25,847 additional users, with youth (aged younger than 20 years) representing 23.9% of those accessing services (unpublished data).

In Burkina Faso, this emphasis on local ownership and supportive state systems has been critical to meeting the needs of displaced groups. The MSI Ladies model recruits qualified midwives, nurses, or committed women who are fully embedded within communities to provide SRH/FP services and advice (including on STIs and HIV screening and help for GBV) to women in their communities. The model, implemented in partnership with the National Ministry of Health and regional and local authorities in Burkina Faso, has been successful at delivering services in a way that is socially and culturally acceptable, including in Burkina Faso’s Centre North Region, which is home to approximately 400,000 internally displaced persons. This approach has been included alongside a broader package of health system strengthening activities (including support to local authorities and service provision sites), helping to ensure that local, regional, and national governments have the appropriate systems and processes in place to support their continuation.

Nevertheless, there remain significant challenges to strengthening the effective delivery of SRH/FP services for displaced populations. In Ethiopia, key health policy documents omit or are vague about preparing for the delivery of SRH/FP services in the event of a public health emergency and pay little consideration to the needs of displaced populations. In South Sudan, policy documents mention the delivery of SRH/FP services to displaced populations but note a lack of data to assess access and uptake. Focusing on policy change and mechanisms for assessing policy implementation and the effectiveness of services at reaching vulnerable and displaced populations must be prioritized as a critical avenue through which to focus attention on need, marshal resources, and coordinate different actors.

### 2. Ensuring Access to SRH Services for Displaced Populations Requires a Coordinated and Adaptive Approach

In the most stable of circumstances, health systems are complex, with multiple and varying interactions between different components and actors.^17^ Health systems require contextually appropriate and politically informed approaches, with dynamic and adaptive learning critical to addressing the needs of the entirety of the population and ensuring high-quality programming.^17^

Health systems require contextually appropriate and politically informed approaches, with dynamic and adaptive learning critical to addressing the needs of the entire population and ensuring high quality programming.

Under the program, rapid and applied political economy analysis and the Pathways of Change tool^18^ have been cornerstones of implementation. Pathways for Change is an innovative adaptive programming tool designed for use under a payment by results contract. The tool requires users to determine their ultimate goal in response to the problem identified, working backward from there to select annual indicators and quarterly milestones to achieve this. This menu of milestones can be adapted during implementation based on situation and problem analysis, priorities, knowledge, and emerging opportunities and commitments.

These approaches have allowed for integration between development and humanitarian programming, enabling the program to rapidly shift to acute response when a crisis occurs and ensure that vulnerable populations are not left behind. In Ethiopia, for example, implementing partners were able to rapidly adjust their strategies to respond to the evolution of conflict by partnering with Afar and Amhara Regional Health Bureaus to support 50 public-sector delivery sites to provide high-quality SRH services in conflict-affected areas and to displaced populations. More recently, in Ethiopia, emphasis has increased on advocating for the prioritization of SRHR in the MISP to ensure continuity of access during protracted crises and transition periods.

Adaptive programming has also enabled the program to respond more intentionally to climate change as a driver of displacement and explore its impact in SRH/FP access. In South Sudan, the program is supporting the Boma Health Initiative to enable those affected and displaced by recurrent flooding in Rubkona and Aweil East to access oral pills, condoms, injectables, and referrals for long-acting reversible contraceptives through a network of community health workers. In Madagascar, where climate-related disasters have led to an estimated 1.1 million internal displacements between 2008 and 2021,^19^ the program is working with the government to explore the impact of climate change on access to SRH/FP services and the extent to which SRH/FP is integrated effectively into disaster response plans. This effort started with research that explored how health providers and communities were experiencing adverse weather events, how these impacted the ability to provide and access health services (with a specific focus on SRH/FP), and the adequacy of government frameworks in supporting appropriate mitigation and adaptation strategies.

### 3. SRHR Interventions Are Lifesaving and Must Be Rights Based

It is important to recognize that displaced populations are not homogenous. Within these populations, there will be different and nuanced needs and vulnerabilities, for example, persons with disabilities, adolescents, and survivors of GBV. Displaced women and girls can have varying levels of knowledge, family size preferences, cultural beliefs around different elements of SRHR, and experiences of trying to access care. The family and community structures surrounding women and girls, as well as the legal frameworks in which they are living, will also differ significantly depending on context, with subsequent impacts on access.^20^

In Ethiopia, a strong emphasis was included on raising awareness and knowledge around issues, such as social violence and GBV, as well as the package of integrated SRH services to address such challenges, with a key focus on including men and boys in health talks. Psychosocial support from trained health professionals was provided, reaching 1,442 people in the final quarter of 2022 in Afar and Amhara.

However, while the needs of displaced women for SRH services is acute, it is important that access to SRH is rights based. As Daigle and Spencer noted, “the impetus to act quickly [can result] in health advice from providers that is based not on patients’ autonomy but rather on underlying assumptions that crisis-affected people should not have (more) children” with little focus on wider sexual health.^21^

Vulnerable communities’ access can also be limited by stigma and negative attitudes toward SRHR from service providers and community members alike, language barriers, lack of health care provider understanding of the specific needs of displaced populations, lack of privacy at service delivery points, and mistrust among displaced communities about the intentions of SRHR policies and programs. Program managers must make complex choices and innovate to address these needs within existing packages of interventions. For example, in Sudan, data enumerators and facility representatives paused the administration of client exit interviews (a key method of securing feedback from clients on the quality, accessibility, and affordability of services) until they had addressed challenges in language and issues of confidentiality caused by the cramped nature of the displacement camps. In the Segou and Tombouctou regions in Mali, MSI’s intention to reach displaced persons was often frustrated due to the struggle of mobile teams to identify those who were displaced from the more general host population and the volatile security situation in areas. To address this, MSI Mali is now working with the local Social Development and Solidarity Economy Department to identify areas with internally displaced persons and train community relays and peer educators to conduct awareness-raising activities. This approach is already yielding promising results in these 2 challenging regions since it was implemented at the end of 2022.

In humanitarian settings, the MISP for SRH developed by the Inter-Agency Working Group on Reproductive Health in Crises provides guidance on the minimum services that should be provided, including contraceptive methods (e.g., condoms, oral and injectable contraceptives, intrauterine devices, and implants); information, education, and communication materials; and contraceptive counseling. The MISP also outlines fundamental principles for such programming, including working in respectful partnership, providing accessible information and choice, recognizing users’ autonomy, and promoting equity.^22^

In Ethiopia, most service providers had not taken an integrated approach to providing SRH services at the primary health care level within conflict settings. Public health officers and clinicians were trained in the MISP, after which service monitoring data for outreach and static facilities showed a more integrated service approach. Providers also focused on ensuring privacy and confidentiality and enrolled health extension workers to engage with community members in and around camps for internally displaced persons. In these engagements, health extension workers facilitate discussions to help them understand the specific needs of the communities they service and ensure that potential clients understand what SRH services are available.

### 4. Self-Care Has the Potential to Be a “Game-Changing” Intervention to Increase Equitable Access to SRH/FP Care

Self-care interventions include (but are not limited to) self-testing for STIs, pre-exposure prophylaxis for HIV prevention, self-managed contraceptives, and postabortion care.^23^ Self-administered injectable contraception (DMPA-SC) has been integrated into national policies and guidelines in countries like Madagascar. In South Sudan, as part of the ongoing National Family Planning Policy review process, self-care modalities have been highlighted as 1 of the key strategies to be included. In other countries, SRH/FP counseling services have been used as an avenue to provide users with access to self-care and other methods suitable to their lifestyle and context. However, as noted by Tran et al., “self-care does not eliminate the need for provider-led facility-based care” and must be rooted within a wider community-centered package of services.^24^

In May 2023, the Inter-Agency Working Group on Reproductive Health in Crises released a new global assessment report on self-care for SRH in humanitarian and fragile settings.^25^ The report findings show that humanitarian, fragile, and stable settings face similar barriers to advancing self-care, including hesitancy to support self-care among a broad set of stakeholders, inadequate financing, and supply chain and measurement challenges. However, new ways of working are needed to make self-care a reality in these settings, including context-specific and user-centered programs, empathy for and trust in self-care users, and new frameworks for measuring impact and quality. Understanding how to ensure better self-care interventions are context specific and user informed is a critical gap to fill, particularly if governments, other partners, and providers are able to ensure high-quality and accountable services that protect the rights of individuals. To this end, a study on the provision of DMPA-SC is underway to understand the feasibility and acceptability of the self-injection among women with low literacy levels in South Sudan’s Awiel East County, documenting sociodemographic factors, experiences, and user preferences that are associated with continuation of use. This study also involves consultations with local leadership, community members, policymakers, and technical experts to understand the programmatic implications of self-injected contraceptive use in a context like rural South Sudan. The results of this study will be available in late 2023 or early 2024.

New ways of working are needed to make self-care a reality in these settings, including context-specific and user-centered programs, empathy for and trust in self-care users, and new frameworks for measuring impact and quality.

## CONCLUSION

The number of individuals migrating both within and across borders will continue to increase, especially as the impacts of climate change, fragile economic systems, and conflict worsen. More needs to be done and published to understand how SRH services are best integrated into country-owned systems to ensure the effective provision of services during humanitarian and protracted crises and recovery phases, as well as for displaced persons in stable host settings. Interventions that combine humanitarian and development programming can support more resilient health systems while recognizing and prioritizing the needs of vulnerable population groups, including displaced persons, living in crisis, transitory, and stable settings.

## Notes

**Peer Reviewed**

**First published online:** August 18, 2023.

## References

[1] International Organization for Migration (IOM). *World Migration Report*. IOM; 2022. Accessed July 28, 2023. https://publications.iom.int/books/world-migration-report-2022

[2] United Nations Department of Economic and Social Affairs (UN). Population Division. *Cross-National Comparisons of Internal Migration: An Update on Global Patterns and Trends*. UN; 2013. Accessed, July 28, 2023. https://www.un.org/en/development/desa/population/publications/pdf/technical/TP2013-1.pdf

[3] United Nations Human Rights Office of the High Commissioner (UNHCR). *Report on the Impact of Climate Change and the Protection of Human Rights of Migrants*. UNHCR; 2022. Accessed July 28, 2023. https://www.ohchr.org/en/calls-for-input/2022/report-impact-climate-change-and-protection-human-rights-migrants

[4] Clement V, Rigaud KK, de Sherbinin A, et al. *Groundswell Report Part 2: Acting on Internal Climate Migration*. World Bank; 2022. Accessed July 28, 2023. https://openknowledge.worldbank.org/handle/10986/36248

[5] Egli-Gany D, Aftab W, Hawkes S, et al. The social and structural determinants of sexual and reproductive health and rights in migrants and refugees: a systematic review of reviews. East Mediterr Health J. 2021;27(12):1203–1213. 10.26719/emhj.20.101. 35137389 PMC7616978

[6] Global Humanitarian Overview 2023. United Nations Office for the Coordination of Humanitarian Affairs. Accessed July 28, 2023. https://humanitarianaction.info/gho2023

[7] Chen YYB. International migrants’ right to sexual and reproductive health care. Int J Gynaecol Obstet. 2022;157(1):210–215. 10.1002/ijgo.14149. 35187657

[8] Inter-Agency Working Group on Reproductive Health in Crises (IAWG). *Inter-Agency Field Manual on Reproductive Health in Humanitarian Settings*. IAWG; 2018. Accessed August 6, 2023. https://iawgfieldmanual.com/manual 26203479

[9] Sphere Association. *The Sphere Handbook: Humanitarian Charter and Minimum Standards in Humanitarian Response*. 4th ed. Sphere Association; 2018. Accessed August 6, 2023. https://www.spherestandards.org/handbook/

[10] International Planned Parenthood Federation (IPPF) with Inter-Agency Working Group on Reproductive Health in Crises. Training Modules on Sexual and Reproductive Health in Emergencies: An Introduction to the Minimum Initial Service Package (MISP) For Sexual and Reproductive Health (SRH). January 29, 2023. Accessed July 28, 2023. https://iawg.net/resources/training-modules-on-sexual-and-reproductive-health-in-emergencies-an-introduction-to-the-minimum-initial-service-package-misp-for-sexual-and-reproductive-health-srh

[11] United Nations Human Rights Office of the High Commissioner (UNHCR). *2021 Annual Public Health Global Review*. UNHCR; 2021. Accessed July 28, 2023. https://www.unhcr.org/media/40241

[12] Hadgkiss EJ, Renzaho AMN. The physical health status, service utilisation and barriers to accessing care for asylum seekers residing in the community: a systematic review of the literature. Aust Health Rev. 2014;38(2):142–159. 10.1071/AH13113. 24679338

[13] High-Impact Practices in Family Planning (HIPs). *Family Planning in Humanitarian Settings: A Strategic Planning Guide*. *Family Planning 2020*. HIPs; 2020. Accessed July 28, 2023. https://www.fphighimpactpractices.org/guides/family-planning-in-humanitarian-settings/

[14] Ready to Save Lives: Sexual and Reproductive Health Care in Emergencies Toolkit. FP2030. Accessed July 28, 2023. https://fp2030.org/srh-toolkit

[15] Bukuluki P, Okwii M, Hoffmann K, Pavin M. *South Sudan Social Norms Assessment*. USAID/Momentum Integrated Health Resilience; 2022. Accessed July 28, 2023. https://usaidmomentum.org/app/uploads/2022/03/MOMENTUM-IHR-South-Sudan-Social-Norms-Assmt-01-21-22.pdf

[16] Couple-years of protection. U.S. Agency for International Development. Accessed July 28, 2023. https://www.usaid.gov/global-health/health-areas/family-planning/couple-years-protection-cyp

[17] Health systems thinking. Options. Accessed July 28, 2023. https://options.co.uk/expertise/health-systems-thinking

[18] Bandali S, Style S, Thiam L, Omar OA, Sabino A, Hukin E. Pathways of change for achieving sustainability results: a tool to facilitate adaptive programming. Glob Public Health. 2022;17(3):457–468. 10.1080/17441692.2020.1868016. 33406002

[19] Country profile Madagascar. Internal Displacement Monitoring Centre. Accessed July 28, 2023. https://www.internal-displacement.org/countries/madagascar#:∼:text=Climate%20change%20impacts%20combined%20with,affected%20by%20drought%20in%202021

[20] Nabulsi D, Abou Saad M, Ismail H, et al. Minimum initial service package (MISP) for sexual and reproductive health for women in a displacement setting: a narrative review on the Syrian refugee crisis in Lebanon. Reprod Health. 2021;18(1):58. 10.1186/s12978-021-01108-9. 33685476 PMC7938550

[21] Daigle M, Spencer A. *Reproductive Justice, Sexual Rights and Bodily Autonomy in Humanitarian Action: What a Justice Lens Brings to Crisis Response*. Humanitarian Policy Group Working Paper. Overseas Development Institute; 2022. Accessed July 28, 2023. https://cdn.odi.org/media/documents/HPG_USAID_SRHR_final_OU8bJbf.pdf

[22] Inter-Agency Working Group on Reproductive Health in Crises (IAWG). *Quick Reference for the Minimum Initial Service Package for Sexual and Reproductive Health*. IAWG. Updated May 16, 2022. Accessed July 28, 2023. https://iawg.net/resources/misp-reference

[23] Logie CH, Khoshnood K, Okumu M, et al. Self care interventions could advance sexual and reproductive health in humanitarian settings. BMJ. 2019;365:l1083. 10.1136/bmj.l1083. 30936067 PMC6441869

[24] Tran NT, Tappis H, Moon P, Christofield M, Dawson A. Sexual and reproductive health self-care in humanitarian and fragile settings: where should we start? Confl Health. 2021;15(1):22. 10.1186/s13031-021-00358-5. 33827633 PMC8024937

[25] Inter-Agency Working Group on Reproductive Health in Crises (IAWG). *Self-Care for Sexual and Reproductive Health in Humanitarian and Fragile Settings: Barriers, Opportunities and Lessons Learned*. IAWG; 2023. Accessed July 28, 2023. https://iawg.net/resources/self-care-srh-humanitarian-fragile-settings-barriers-opportunities-and-lessons-learned

